# Incidental Finding of an Aorto-Right Atrial Fistula in a Patient Undergoing Repair of a Sinus of Valsalva Aneurysm

**DOI:** 10.3389/fmed.2017.00095

**Published:** 2017-06-30

**Authors:** Byron R. Rosero-Britton, Anthony Nguyen, Ibrahim Warsame, Muhammad Shabsigh, Luke Dong, James Wolfe, Bryan Whitson, Michael Essandoh

**Affiliations:** ^1^The Ohio State University College of Medicine, Columbus, OH, United States

**Keywords:** sinus of Valsalva aneurysm, right atrium, transesophageal echocardiography, sinus of Valsalva-to-right atrial fistula, computed tomography angiography

## Abstract

A sinus of Valsalva aneurysm is a rare malformation of the aortic root that can fistulize to another cardiac structure such as the right atrium. Although transthoracic echocardiography and computed tomography angiography have demonstrated utility for the diagnosis of a sinus of Valsalva-to-right atrial fistula, there are few cases where a misdiagnosis may occur. Intraoperative transesophageal echocardiography may be an essential imaging tool for the diagnosis and management of incidental findings such as a sinus of Valsalva-to-right atrial fistula during cardiac surgery and should be used routinely.

## Introduction

A sinus of Valsalva aneurysm (SOVA) is a rare cardiac anomaly where dilation of the wall of the aortic root area between the aortic valve annulus and the sinotubular ridge develops and is often associated with numerous cardiac complications that dictate the acuity and severity of its clinical presentation (1–8). Surgical intervention has shown promising outcomes with 10-year survival ranging between 63 and 100%, but given the often-urgent nature of the presentation of a SOVA, accurate diagnosis and anticipation of associated complications is imperative to guide appropriate, and potentially life-saving, therapy (9, 10).

The authors present the case of a patient undergoing a SOVA repair in which an aorto-right atrial fistula was incidentally identified on intraoperative transesophageal echocardiography (TEE) examination; the presence of the fistula prompted a change in the intraoperative management of the patient.

## Case Presentation

A 57-year-old male with a past medical history of hypertension, myocardial infarction, hyperlipidemia, and sepsis was found by his physician to have a murmur during a visit for severe exertional dyspnea. A transthoracic echocardiogram (TTE) showed a large SOVA located at the non-coronary cusp, moderate enlargement of the right atrium (RA) and the right ventricle (RV), mild systolic dysfunction of the left ventricle, and no echocardiographic evidence of valvular pathology or intracardiac shunts. A computed tomography angiogram (CTA) was obtained to further characterize the SOVA and revealed a 2.2 cm × 1.6 cm × 2.3 cm saccular aneurysm herniating from the non-coronary cusp into the RA, in addition to a dilated RA and a dilated RV. The CTA further demonstrated the following: complete opacification of the RA, the RV, and the aortic root by the intravenous contrast agent, and extensive reflux of contrast into the inferior vena cava and the hepatic veins during systole that was suggestive of significant tricuspid valve insufficiency or a large left-to-right shunt. The likelihood of an intracardiac shunt was high considering the absence of tricuspid insufficiency on the TTE examination; however, further cardiac imaging was not considered since TEE monitoring was being employed intraoperatively. A percutaneous coronary angiogram was also performed, which revealed mild non-obstructive atherosclerotic disease.

**Figure 1:**
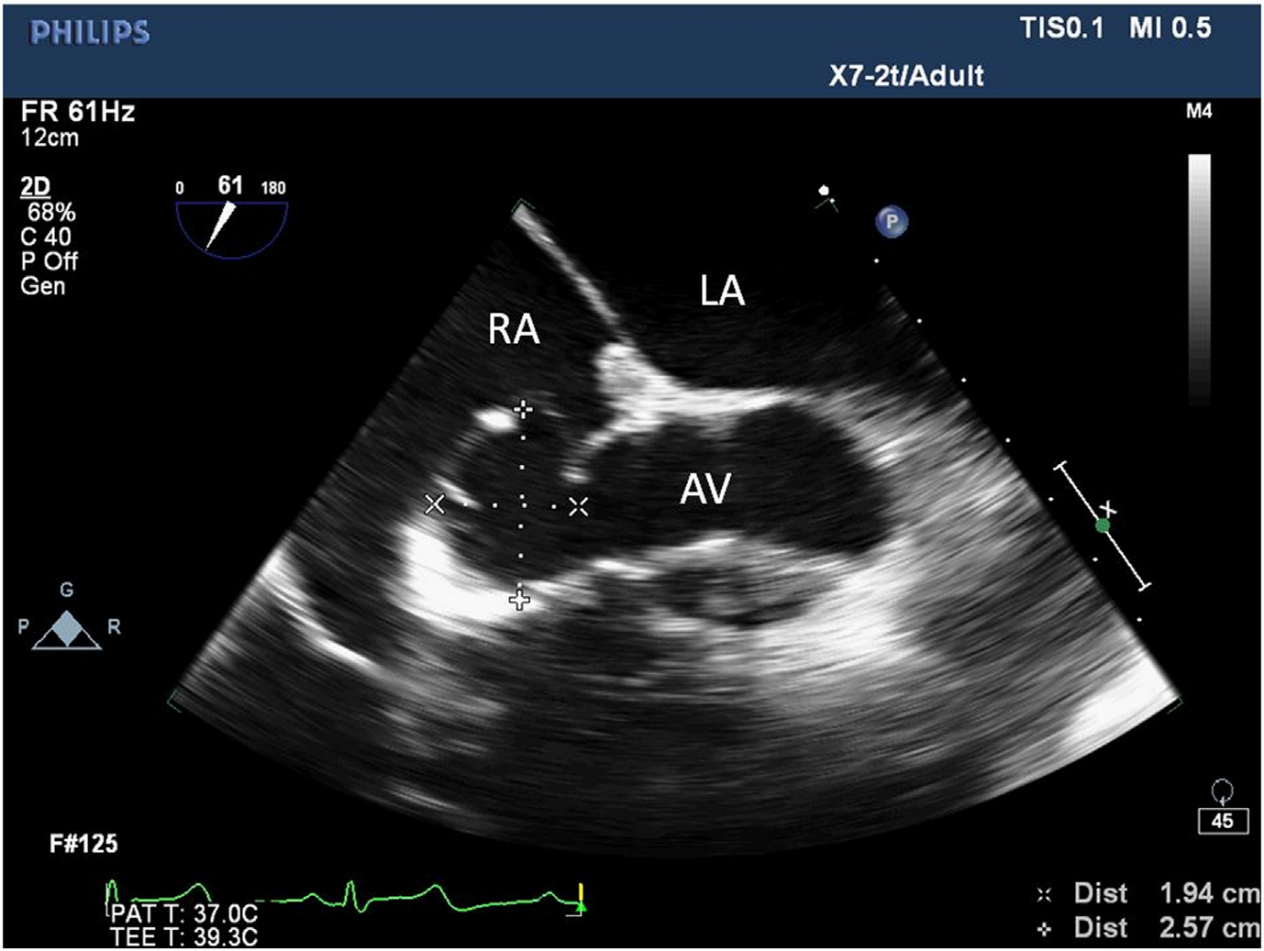
Two-dimensional echocardiogram short-axis view of the aortic valve showing an aneurysm at the non-coronary sinus of Valsalva measuring 1.94 cm × 2.57 cm. LA, left atrium; RA, right atrium; AV, aortic valve.

**Figure 2:**
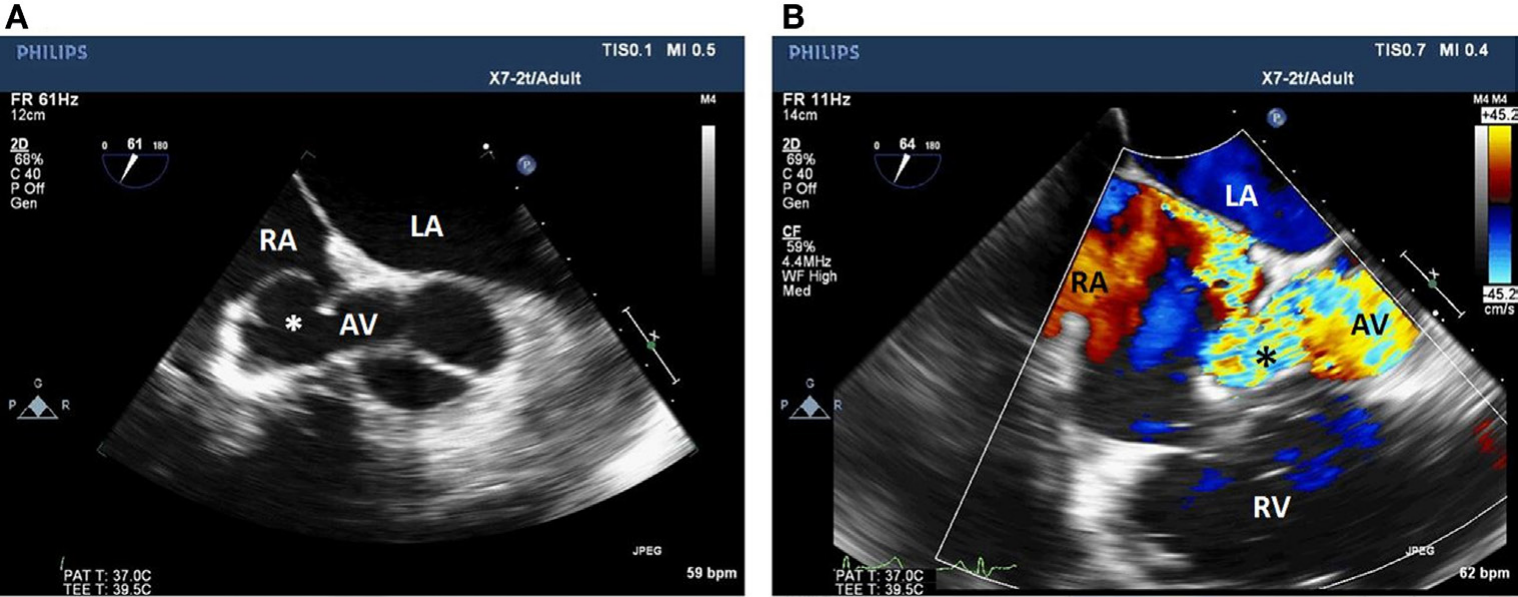
Two-dimensional echocardiogram short-axis view of the aortic valve **(A)** with color flow Doppler **(B)** showing a fistula between the non-coronary sinus of Valsalva aneurysm and the RA. LA, left atrium; RA, right atrium; AV, aortic valve; Asterix, sinus of Valsalva aneurysm; RV, right ventricle.

**Figure 3:**
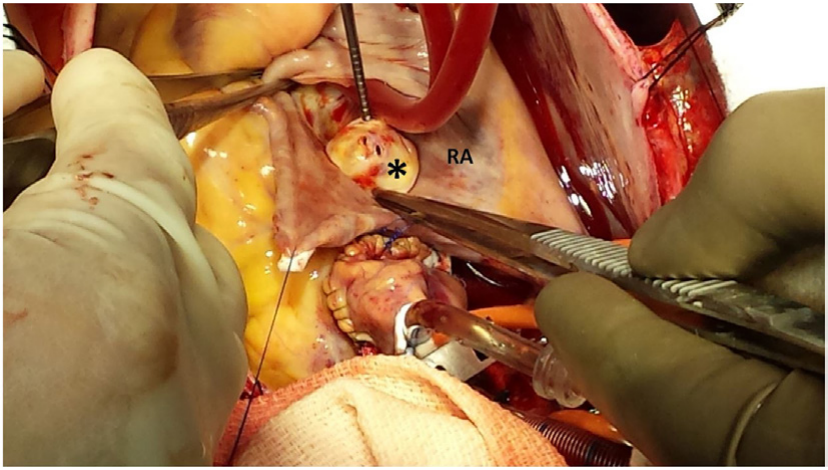
Image demonstrating the non-coronary sinus of Valsalva aneurysm communicating with the RA. RA, right atrium; Asterix, sinus of Valsalva aneurysm.

**Figure 4:**
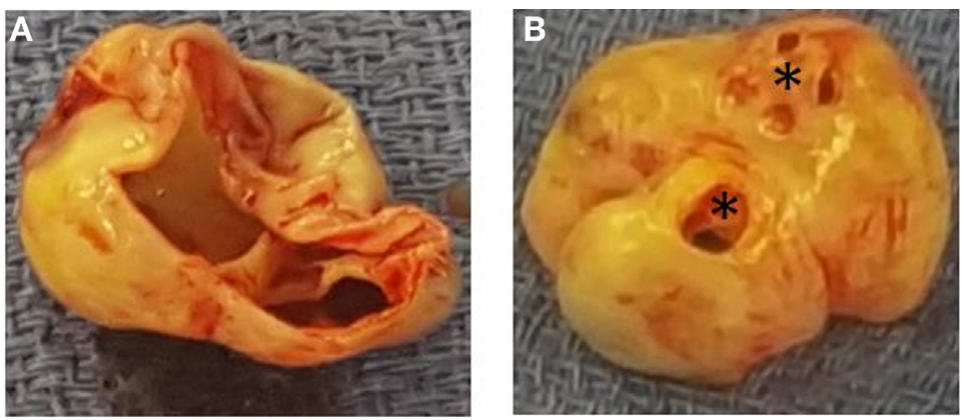
Panel demonstrating the non-coronary sinus of Valsalva aneurysm form the perspective of the aortic root **(A)** and the perspective of the right atrium **(B)**. Asterix, sinus of Valsalva aneurysm-to-right atrial fistulas.

The patient was subsequently scheduled for repair of the SOVA. A left radial intra-arterial catheter was inserted for hemodynamic monitoring prior to the induction of general anesthesia and endotracheal intubation. Post-intubation, a TEE probe was inserted to guide central venous catheter placement and hemodynamic monitoring. The TEE demonstrated a 1.9 cm × 2.6 cm saccular aneurysm at the level of the non-coronary sinus, which communicated with the RA upon color flow Doppler analysis (Figures 1 and 2; Videos S1 and S2 in Supplementary Material), a finding not apparent on the pre-operative CTA or TTE. Substantial communication between the ascending aorta and the RA was identified and the decision was made to avoid placing a pulmonary arterial catheter, which potentially could access the SOVA–RA fistula (Figures 3 and 4) and cause aortic injury. Cardiopulmonary bypass was then instituted after adequate systemic anticoagulation with heparin. The orifice of the SOVA was repaired with a bovine pericardial patch successfully. Afterward, the fistula tract was accessed using a right atriotomy and was excised and oversewn. The patient tolerated the procedure well and had an uneventful recovery.

## Discussion

A SOVA is a rare cardiac malformation involving the aortic root. It predominantly originates from the right coronary cusp (39–89%), followed by the non-coronary cusp, and rarely from the left coronary cusp (3–6, 9, 11–20). Diagnosis of a SOVA often warrants further evaluation, as its presence is often associated with various cardiac sequelae such as aneurysmal rupture, aneurysmal fistulization, ventricular septal defect, RV outflow tract obstruction, and aortic valve dysfunction (3).

Transthoracic echocardiography, the initial diagnostic study performed in the presented case, often provides sufficient information regarding the presence, location, and complicating cardiac lesions associated with a SOVA. Although TTE appropriately identified the presence and origin of a SOVA, it failed to discover the communicating tract between the SOVA and the RA. Furthermore, the TTE did not indicate any evidence of aortic insufficiency or any other valvular pathology, as initially anticipated following the discovery of the murmur, which in retrospect was due to blood flow from the aorta root to the RA. In a recent report by Cheng et al., TTE provided a diagnostic sensitivity, specificity, and accuracy in the identification of SOVA of 93.9, 99.9, and 99.8%, respectively (3). Moreover, the authors reported that TTE could be relied upon to accurately diagnose the presence of aneurysmal rupture in most cases (97.5%).

Multimodal cardiac imaging, in the form of CTA and cardiac magnetic resonance imaging (CMRI), can complement echocardiography. Both CTA and CMRI offer excellent anatomical depiction of a SOVA and may help identify fistulous tracts (21, 22). The CTA in the case presented, however, did not identify the SOVA to RA fistula due to concomitant opacification of the aorta and the RA by the radiographic contrast. The reflux of contrast material into the inferior vena cava and hepatic venous system was due to the fistula (23). Retrograde aortography previously served as an invaluable diagnostic tool for patients with suspected SOVA and was relied upon particularly prior to the advancements in echocardiography (24). Considering that contrast is administered in the aorta immediately before imaging, the presence of a fistula to the RA can be rapidly identified with a retrograde aortogram. In the presented case, direct ostial access of the right coronary and left coronary circulation during the cardiac catheterization precluded any assessment of the non-coronary sinus, thus resulting in the failure to identify the fistulous tract.

Finding an aorto-RA fistula early during the operative course holds major implications for both the cardiac anesthesiologist and the cardiothoracic surgeon. During pulmonary artery catheter placement, inadvertent passage of the catheter through the fistulous tract may cause aneurysmal rupture and hemodynamic compromise. Furthermore, identification of such findings may alter surgical management and should be communicated as quickly as possible to accommodate additional procedures as needed.

In the case presented, despite undergoing a comprehensive cardiac work-up prior to surgery inclusive of a TTE, a CTA, and a coronary angiogram, the fistulous tract between the non-coronary cusp and the RA was clearly not identified, and highlights the utility of, and further advocates for early intraoperative application of TEE for the elucidation of overall cardiac pathophysiology in patients undergoing SOVA repair.

## Summary

Transesophageal echocardiography is a useful imaging modality that can provide clarity and insight into often challenging cardiac presentations. It is incumbent upon the cardiac anesthesiologist to display medical acumen and effectively utilize this imaging modality. Although standard practice does not dictate early application of TEE during the operative course, we recommend that TEE be utilized immediately post-induction to detect any previously unforeseen cardiac pathology and better guide both anesthetic and surgical care in patients undergoing SOVA repair.

## Ethics Statement

Consent was obtained for publication of this case report.

## Author Contributions

All authors contributed equally to the creation of this manuscript.

## Conflict of Interest Statement

The authors declare that the research was conducted in the absence of any commercial or financial relationships that could be construed as a potential conflict of interest.

## Supplementary Material

Video S1 Two-dimensional echocardiogram right ventricular inflow–outflow view demonstrating a non-coronary sinus of Valsalva aneurysm.

Video S2 Two-dimensional echocardiogram right ventricular inflow–outflow view with color flow Doppler demonstrating a non-coronary sinus of Valsalva aneurysm-to-right atrial fistula.
